# A novel approach to medicines optimisation post-discharge from hospital: pharmacist-led medicines optimisation clinic

**DOI:** 10.1007/s11096-020-01059-4

**Published:** 2020-06-11

**Authors:** Mohanad Odeh, Claire Scullin, Anita Hogg, Glenda Fleming, Michael G. Scott, James C. McElnay

**Affiliations:** 1 Pharmacy Management and Pharmaceutical Care Innovation Centre, Hashemite University, 13133 Hashemite University, Zarqa, Jordan; 2 Clinical and Practice Research Group, School of Pharmacy, Queen’s University Belfast, 97 Lisburn Road, Belfast, BT9 7BL UK; 3 Medicines Optimisation Innovation Centre (MOIC), Bretten Hall, Northern Health and Social Care Trust, Antrim Site, Antrim, UK

**Keywords:** Cost perspective, Medicines optimisation clinic, Medicine review, Patient care, Pharmacist-led services, Readmission, United Kingdom

## Abstract

*Background* There is a major drive within healthcare to reduce patient readmissions, from patient care and cost perspectives. Pharmacist-led innovations have been demonstrated to enhance patient outcomes. *Objective* To assess the impact of a post-discharge, pharmacist-led medicines optimisation clinic on readmission parameters. Assessment of the economic, clinical and humanistic outcomes were considered. *Setting* Respiratory and cardiology wards in a district general hospital in Northern Ireland. *Method* Randomised, controlled trial. Blinded random sequence generation; a closed envelope-based system, with block randomisation. Adult patients with acute unplanned admission to medical wards subject to inclusion criteria were invited to attend clinic. Analysis was carried out for intention-to-treat and per-protocol perspectives. *Main Outcome Measure* 30-day readmission rate. *Results* Readmission rate reduction at 30 days was 9.6% (*P* = 0.42) and the reduction in multiple readmissions over 180-days was 29.1% (*P* = 0.003) for the intention-to-treat group (*n* = 31) compared to the control group (*n* = 31). Incidence rate ratio for control patients for emergency department visits was 1.65 (95% CI 1.05–2.57, *P* = 0.029) compared with the intention-to-treat group. For unplanned GP consultations the equivalent incident rate ratio was 2.00 (95% CI 1.18–3.58, *P* = 0.02). Benefit to cost ratio in the intention-to-treat and per-protocol groups was 20.72 and 21.85 respectively. Patient Health Related Quality of Life was significantly higher at 30-day (*P* < 0.001), 90-day (*P* < 0.001) and 180-day (*P* = 0.036) time points. A positive impact was also demonstrated in relation to patient beliefs about their medicines and medication adherence. *Conclusion* A pharmacist-led post-discharge medicines optimisation clinic was beneficial from a patient care and cost perspective.

## Impacts on practice

- Inpatient clinical pharmacist-led medicines optimisation services result in significant improvements in the quality and safety of patient care, yielding health gain and economy
- The pharmacist-led medicines optimisation clinic (MOC) model mirrors disease specific outpatient clinics, providing medicines support to post-discharge patients at risk of medicine related problems
- Although only in the pilot phase, this new care model has been shown to have a positive impact on rehospitalisation, cost of care and a range of patient centred humanistic outcome measures

## Introduction

One strategic target in health care systems is to reduce the frequency of unplanned re-hospitalisations, which have progressively increased in rate and cost burden worldwide. Since optimising medicines use is a key process in effective disease management, pharmacists have a significant role to play in patient care after discharge from hospital.

In the United Kingdom £17.4 billion was spent on medicines during 2016/17 [1]. Importantly, however, a high percentage (33–50%) of medicines prescribed for long term conditions are not taken as recommended, with only 16% of newly prescribed medicines taken correctly [2–4]. Further to this, one in eight patients has a medicine related problem [5]. Certain patients are at high risk of having difficulties in managing their medicines when discharged from hospital which can often result in the patient being readmitted [6]. Although care is taken to support patients managing their medicines and medical conditions prior to discharge [7–9], they often face significant challenges when they return home.

It is recognised that performing medication reconciliation alone (10) and isolated discharge planning efforts cannot resolve all problems (11) nor can isolated discharge support (12). Evidence has shown that positive effects are mainly observed when interventions from the discharge planning and post-discharge support perspectives are combined across the hospital-home interface (10, 13). However, further well-designed and carefully conducted studies to improve the effectiveness of healthcare delivery for recently discharged patients are required in this area (10, 14).

In order to perform the present research, a collaborative team developed an outpatient medicines optimisation clinic (MOC), led by clinical pharmacists, to provide support post-discharge to patients deemed to be at risk of medicine related problems. The hypothesis was that patients at risk of medicine related problems, could benefit from outpatient follow-up by hospital based clinical pharmacists, building on the Integrated Medicines Management services [7, 9] provided to hospital inpatients at the study site hospital.

## Aim of the study

The aim was to assess the impact of a pharmacist-led MOC on both patient and health care system outcomes, when delivered to patients at high risk of medicine related problems after a period of hospitalisation.

## Ethics approval

Ethics approval for the study was obtained from the Office for Research Ethics Committees Northern Ireland (IRAS 11/NI/0127)/Clinical Trials.Gov registration no: NCT01534559.

## Method

The study was designed as a parallel group, randomised, controlled trial with 1:1 allocation ratio. Patient recruitment was conducted in the respiratory and cardiology wards of Antrim Area Hospital, a 426-bed district general hospital in Northern Ireland.

Adult patients (≥ 18 years old) who were admitted into the study wards, as acute/unscheduled medical admissions, who met at least one of the inclusion criteria were invited to participate and provide written informed consent (Box 1).
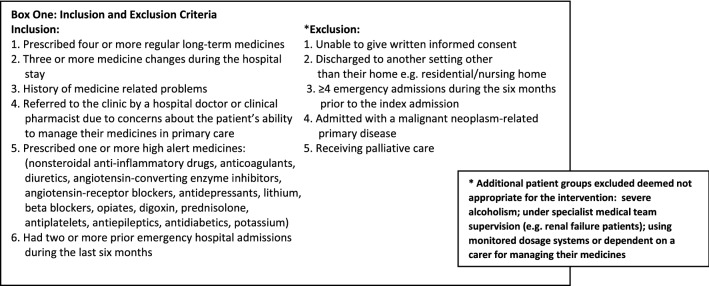


During their hospital stay, recruited patients within both groups completed three self-reported questionnaires: Medication Adherence Report Scale (MARS), Patient Beliefs about Medicines Questionnaire (BMQ) and health-related quality of life (HRQOL) questionnaire (EQ-5D-3L). These questionnaires were mailed to participating patients for self-completion at 30, 90, 180 and 365 days post-discharge. Complete questionnaires were mailed to the researcher for analysis.

This time bound (12 month) pilot study evaluated the impact of the new clinic service. Patients were screened for eligibility in participating wards before being invited to participate. During patient enrolment, concealed allocation to a control or intervention group was achieved through a closed envelope system [10] prepared by an independent investigator. Block randomisation with random block sizes, ensured allocation balance and avoided selection bias by preventing allocation prediction [11]. A sample size calculation was not carried out for this pilot study. Researchers and pharmacists were blind to the block size sequence and randomisation envelopes were unopened until after patient recruitment. Patients were blinded to the primary outcome measure.

The MOC was patient centred, focused on the patients’ needs and was conducted at the study-site hospital within 2 weeks of discharge, in a semi-structured format using a bespoke Medicine Review (clinical; level 3) Questionnaire. The clinical pharmacist ensured that each patient received the following services at the clinic: medicines reconciliation; lab test review; medicines review; general patient education (including medicine purpose, dose, timing, potential side effects); lifestyle advice, as appropriate (e.g. physical activity, diet, weight management, smoking cessation, secondary prevention advice); assessment of medication adherence, disease management advice and self-management advice as required. At the end of the appointment, the clinical pharmacist agreed next steps with the patient, and documented key points on a tailored Take Home Action leaflet for the patient to take away. After this first outpatient clinic visit, based on the individual patient’s needs, he/she was invited to a further follow-up (either at the clinic or by telephone) within 6-8 weeks of discharge. A summary of each review was forwarded to the patient’s GP and appropriate hospital medical consultant, which detailed a list of recommendations from the pharmacist. Control patients received the normal post-discharge care, which did not involve any follow-up by a hospital based clinical pharmacist.

The primary outcome measure was 30-day unplanned readmission to hospital. Secondary outcomes included: readmission within 7, 14, 90, 180 and 365 days, time to hospital readmission and length of hospital stay during first readmission. Unplanned GP consultations (including out-of-hours) and Emergency Department (ED) visits over the study follow-up period were recorded. Information regarding healthcare resource usage was obtained from the hospital and corporate information systems by the researcher. Adherence, beliefs about medicines and HRQOL were assessed at baseline and at 30, 90, 180 and 365 days. Patient satisfaction for the intervention group was assessed after the delivery of the service. Finally, a cost–benefit analysis was performed [12, 13]. Analysis was carried out from hospital perspective over a 1 year follow-up period. All relevant costs were obtained from the corporate department of the hospital.

Data were entered and analysed using SPSS version 24 for intention-to-treat (ITT) and per-protocol (PP) analyses. Alternative software (MedCalc^®^) was utilised when SPSS was not appropriate and standard statistical procedures were applied. Survival analysis using Kaplan–Meier curves [14, 15] was used to examine time to readmission. The difference in frequencies of multiple readmissions, unplanned GP consultations and ED visits were analysed using the Mann–Whitney *U* Test [16], which was also used to test differences in length of hospital stay during the first readmission. Relative risk and number needed to treat were calculated when appropriate [17]. Prediction models for number of multiple readmissions, ED visits and unplanned GP consultations were carried out through the development of a generalised linear model, Poisson regression [18, 19]. All missing data was appropriately recorded in the SPSS database prior to analysis.

## Results

### Patient enrolment

**Fig. 1:**
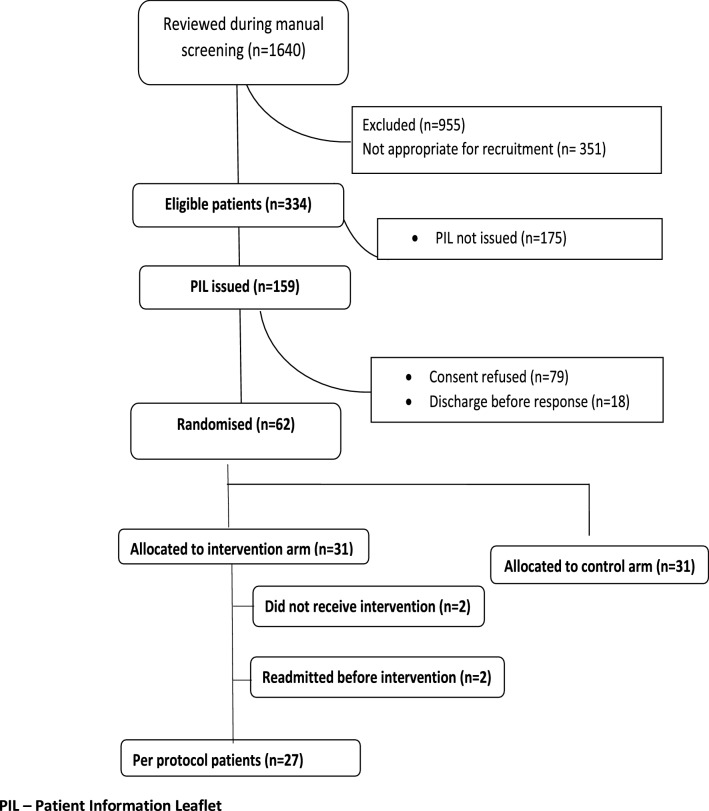
Patient participation flow within the randomised controlled pilot study

Figure 1 illustrates the patient flow for the study. A total of 1640 patients were screened (eligibility ratio was 20.4%). A total of 159 patients were invited to join the study (issued with study details) and 62 patients agreed to participate in this pilot (38.9% of those approached) while 79 patients (49.6%) declined participation after receiving the study information documentation; 18 patients (11.3%) were discharged before they decided.

Table 1 defines the patient characteristics at baseline. There were no statistically significant differences between control and intervention patients.

**Table 1:** Baseline basic characteristics for all randomised patients

Characteristics	Intervention	Control	*P* value
*Gender*			
Male	16	19	0.96
Female	15	12	0.95
Age (Mean years)	67.3	67.6	0.55
*Hospital department*			
Respiratory	22	22	–
Cardiovascular	9	9	–
Index Length of hospital stay (Median interquartile range days)	7 (4–10)	7 (3–13)	0.88
Mean number of prescribed medicines (Standard deviation)	8.95 (3.65)	9.35 (3.69)	0.66
Mean number of high alerts prescribed medicines (Standard deviation)	2.65 (1.84)	2.70 (1.59)	0.91
Smoking (%)	9 (29.0%)	7 (22.6%)	0.56

In total 13 patients attended the MOC once and 7 patients attended twice. In addition 4 patients attended the MOC once with 1 follow-up telephone call, 3 patients received a phone call and the remaining 4 intervention patients received no intervention but were included in the ITT approach for analysis

### Primary outcome: 30-day readmission rate

**Fig. 2:**
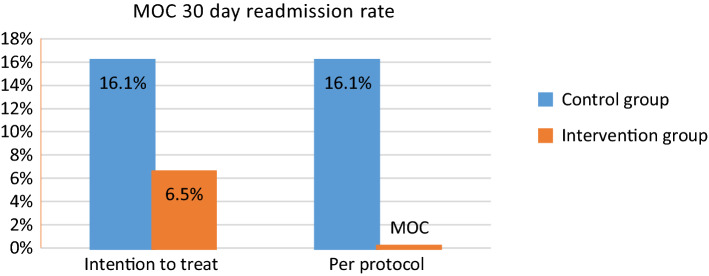
Readmission rate at 30-days post-discharge (control: n = 31; ITT: n = 31; PP: n = 27)

No patients who received the clinic intervention were readmitted within 30-days, i.e. a 16.1% difference from the control group (PP analysis, Fig. 2). Due to the small sample size, this difference just failed to reach statistical significance (*P* = 0.055). Using the ITT approach, the 30-day readmission rate for the intervention group was 6.5% versus the 16.1% readmission rate for the control group (*P* = 0.42). The relative risk of 30-day readmissions was 0.40 (95% CI 0.084–1.91) in the ITT approach; it was 0.10 (95% CI 0.006–1.8) in the PP approach. The number of patients needed to treat (to receive intervention) to avoid one hospitalisation was 6.49 patients (95% CI 3.2–144.2).

### Readmission rates at other time intervals

Table 2 lists readmission rates at 7, 14, 90, 180 and 365 days. The difference in readmission rates between control and the PP group was highest at the 180-day interval, i.e. 23.4% (*P* = 0.055) which resulted in a reduction of risk of readmission by 56% (RR = 0.44, 95% CI 0.18–1.08). The number of patients needed to treat (to prevent one hospitalisation within 180 days) was 4.27 patients (95% CI 2.15–311.80).

**Table 2:** Readmission rates at other time intervals

Time interval	Control *n* = 31	ITT (*n* = 31)		PP (*n* = 27)	
	Number of readmitted patients (%)	Number of readmitted patients (%)	*P* value (Readmission rate difference)	Number of readmitted patients (%)	*P* value (Readmission rate difference)
7-day	2 (6.5%)	0 (0.0%)	0.49 (6.5%)	0 (0.0%)	0.49 (6.5%)
14-day	4 (12.9%)	0 (0.0%)	0.11 (12.9%)	0 (0.0%)	0.11 (12.9%)
90-day	8 (25.8%)	6 (19.4%)	0.75 (6.4%)	4 (14.8%)	0.45 (11.0%)
180-day	13 (41.9%)	7 (22.6%)	0.10 (19.3%)	5 (18.5%)	0.055 (23.4%)
365-day	18 (58.1%)	13 (41.9%)	0.9 (16.2)	10 (37.0%)	0.11 (21.1%)

### Multiple readmissions

Data on multiple readmissions are presented in Table 3 for the 90, 180 and 365 day intervals. Data for the 180 day interval, as an illustrative sample, were as follows: No patients within the PP group (*n* = 27) had multiple readmissions during the 180-day follow-up period. The difference between the control group and the ITT group at 180 days was 29.1% (*P* = 0.003). The risk of multiple readmissions was significantly reduced by 90% (RR = 0.10, 95% CI 0.014–0.74; *P* = 0.024). For the PP group, the observed multiple readmission rate difference was 32.3% (*P* = 0.001). Relative risk reduction was 94.6% (RR = 0.054, 95% CI 0.003–0.89; *P* = 0.04). The number needed to treat to prevent multiple readmissions for the PP group was 3.22 patients (95% CI 2.04–7.67).

**Table 3:** Multiple readmissions of patients

	Control (n = 31)	ITT (n = 31)	*P* value	PP (n = 27)	*P* value
*90-day interval*					
n patientswho had multiple readmissions (%)^a^ [n readmitted patients]	6 (19.4%) [8]	1 (3.2%) [6]	0.104	0 [4]	0.026*
Relative Risk (95% CI)	1	0.17 (0.021–1.31)		0.088 (0.005–1.49)	
Number needed to treat (95% CI)		6.20 (3.19–111.89)		5.40 (2.93–34.09)	
Total n of readmissions^b^ Median (Interquartile range)	26 (8 + 18) 2.5 (1.25–3.00)	7 (6 + 1) 1.0 (1.0–1.0)	0.043*	4 (4 + 0) 1.0 (1.0–1.0)	0.048*
Control Incidence Rate Ratio^c^ (95% CI) [Model fit, Omnibus Test *P* value]	–	2.79 (1.21–6.42) [0.009*]	0.016*	3.25 (1.13–9.31) [0.012*]	0.028*
*180-day interval*					
n patients who had multiple readmissions (%)^a^ [n readmitted patients]	10 (32.3%) [44]	1 (3.2%) [7]	0.003*	0 [5]	0.001*
Relative Risk (95% CI)	1	0.10 (0.014–0.74) *		0.054 (0.003–0.89) *	
Number needed to treat (95% CI)	–	3.44 (2.15–8.74)		3.22 (2.04–7.67)	
Total n of readmissions^b^ Median (Interquartile range)	36 (13 + 23) 2.0 (1.50–3.00)	8 (7 + 1) 1.0 (1.0–1.0)	0.011*	5 (5 + 0) 1.0 (1.0-1.0)	0.010*
Control Incidence Rate Ratio^c^ (95% CI) [Model fit, Omnibus Test *P* value]	–	2.42 (1.13–5.21) [0.014*]	0.024*	2.77 (1.09–7.06) [0.016*]	0.033*
*365-day interval*					
n patients who had multiple readmissions (%)^a^ [n readmitted patients]	11 (35.5%) [13]	4 (12.9%) [44]	0.038*	3 (11.1%) [41]	0.030*
Relative Risk (95% CI)	1	0.36 (0.13–1.02)		0.31 (0.097–1.01)	
Number needed to treat (95% CI)		4.43 (2.32–49.64)		4.10 (2.20–31.21)	
Total n of readmissions^b^ Median (Interquartile range)	42 (18 + 24) 2.0 (1.0–3.0)	17 (13 + 4) 1 (1.0–2.0)	0.068	13 (10 + 3) 1 (1.0–2.0)	0.089
Control Incidence Rate Ratio^c^ (95% CI) [Model fit, Omnibus Test *P* value]		1.78 (1.02–3.13) [0.037*]	0.044*	1.80 (0.96–3.34) [0.053]	0.065

^a^Fisher’s Exact Test or Chi square test. *statistically significant at 0.05 level

^b^Total of 1st readmission occasion plus subsequent readmissions, Mann–Whitney U test

^c^Generalized linear model (Poisson regression), when intervention arm is the reference value (1). Dependent variable (no of readmissions)

According to the Poisson regression presented in Table 3, at the 180-day interval the number of readmissions for the control group will be 2.42 times greater than the ITT group, Incidence Rate Ratio (IRR) = 2.42 (95% CI 1.13–5.21, *P* = 0.024). The regression model was statistically significant (Omnibus Test, *P* = 0.014). The IRR for the PP population was 2.77 (95% CI 1.09–7.06; *P* = 0.033); the predicted model was statistically significant (Omnibus Test, *P* = 0.016).

### Length of hospital stay during the first readmission

Distribution of median (interquartile range) length of hospital stay during the first readmission was the same (*P* > 0.05) across the control and intervention groups, i.e. median of 5 days in all groups [Control = 5 (1.8–10.8); ITT = 5 (2.0–8.5); PP 5 (2.0–10.3)].

### Time to readmission

**Fig. 3:**
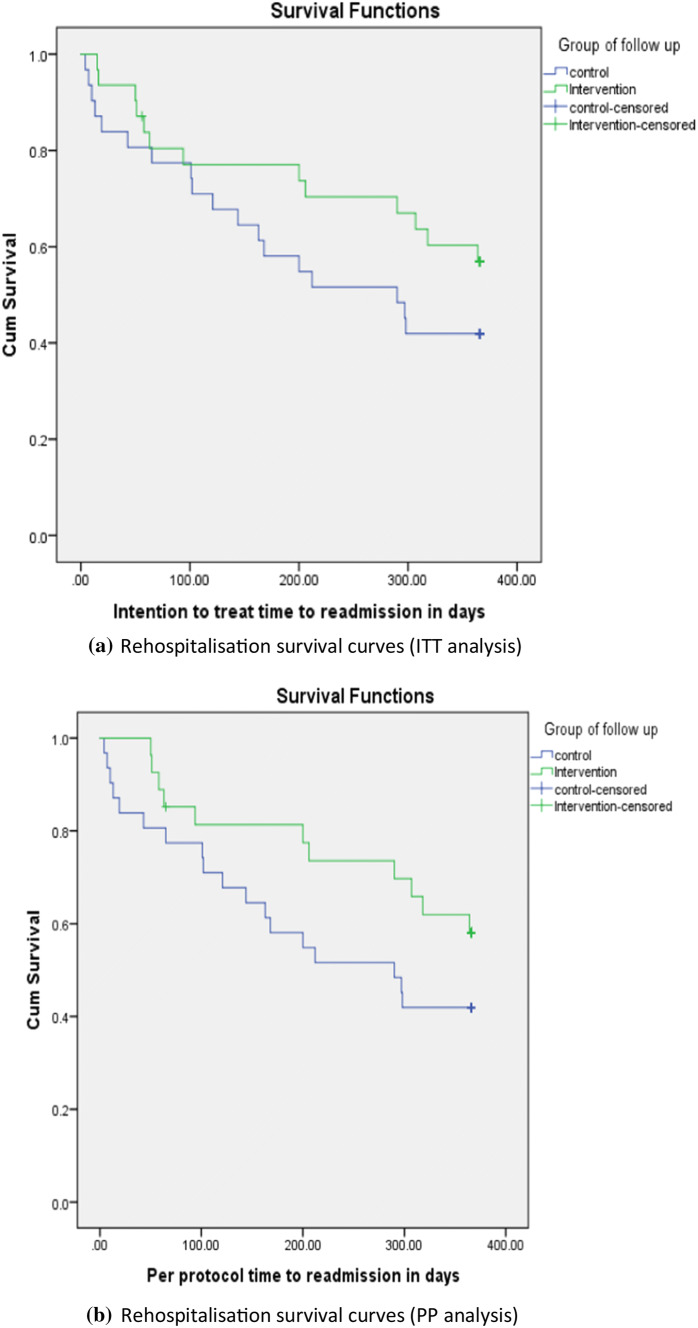
a Rehospitalisation survival curves (ITT analysis), b: Rehospitalisation survival curves (PP analysis)

Figure 3a presents the 365-day Kaplan–Meier survival curve of readmission events after the index discharge. The estimated marginal mean time to readmission for the ITT group was 276.3 days (95% CI 229.8–322.9), while it was 226.3 days (95% CI 176.6–276) for the control group. Nonetheless, this 50.0-day difference was not statistically significant (log rank *P* value was 0.19). When using the PP analysis, intervention patients who attended the MOC showed slightly longer ‘survival times’ prior to readmission, as the estimated marginal mean was 289.7 days (95% CI 245.0–334.4). However, when compared with the control group the difference of 63.4 days was not statistically significant (log rank *P* value: 0.15). (Figure 3b)

### ED visits and unplanned GP consultations

The intervention resulted in a lower total number of emergency department visits (Table 4); 31 vs 51 in the ITT group and 21 vs 51 in the PP group, however, both cases did not reach statistical significance (*P* > 0.05).

**Table 4:** ED visits and unplanned GP consultations

	Control (n = 31)	Intervention (n = 31)	*P* value	Per protocol (n = 27)	*P* value
*Emergency department visit*					
n patients (%)^a^	17 (54.8%)	17 (54.8%)	–	14 (51.8%)	0.82
Relative risk (95% CI)		–		0.95 (0.58–1.53)	
Number needed to treat (95% CI)				–	
Total n of visits^b^ (Median) [Interquartile range]	51 (2.0) [1.0–4.0]	31 (1.0) [1.0–2.5]	0.59	21 (1.0) [1.0–2.0]	0.39
Incidence rate ratio^c^ (95% CI) [Model fit, Omnibus Test *P* value]	–	1.65 (1.05–2.57) [0.026*]	0.029*	2.12 (1.27–3.52) [0.003*]	0.004*
*Unplanned GP consultation*					
n patients (%)^a^	15 (48.4%)	8 (25.8%)	0.066	6 (22.2%)	0.039*
Relative risk (95% CI)	1	0.53 (0.27–1.07)		0.46 (0.21–1.02)	
Number needed to treat (95% CI)		Poor benefit		3.82 (2.0 – 43.47)	
Total n of visits^b^ (Median) [Interquartile range]	34 (1.0) [1.0–3.0]	17 (1.5) [1.0–2.75]	0.07	12 (1.0) [1.0–2.0]	0.034*
Incidence rate ratio^c^ (95% CI) [Model fit, Omnibus Test *P* value]		2.00 (1.18–3.58) [0.016*]	0.020*	2.47 (1.28–4.77) [0.004*]	0.007*

^a^Chi square test

^b^Mann–Whitney test

^c^Generalised linear model (Poisson regression), when intervention arm is the reference value (1). Dependent variable (no of ED visits, no of unplanned GP consultations)

*statistically significant at 0.05 level

The clinic intervention resulted in a reduction in the number of unplanned GP consultations. The risk was reduced by 47% (RR = 0.53, 95% CI 0.27–1.07) for the ITT group, and 54% (RR = 0.46, 95% CI 0.21–1.02) for the PP group. The latter risk reduction demonstrated a borderline significant value (*P* = 0.054). The number needed to treat was calculated at 3.82 patients (95% CI 2.00–43.47) in the PP group.

Table 4 also shows that the Incidence Rate Ratio was 1.65 (95% CI = 1.05–2.57; *P* = 0.029) for ED visits using the ITT data. The prediction model had an acceptable fit to the data (Omnibus Test, *P* = 0.026). Results for the PP group were as follows: the IRR was 2.12 (95% CI = 1.27–3.52; *P* = 0.004) and the Omnibus Test was significant (*P* = 0.003). Unplanned GP consultations gave rise to similar positive outcomes resulting from the MOC engagement.

### Economic evaluation of the MOC intervention

Table 5 shows the cost–benefit analysis using both analytical approaches. A positive benefit to cost ratio was exhibited: 20.72 for the ITT group and 21.85 for the PP approach respectively.

**Table 5:** Resource use and cost–benefit analysis using the median data in the study

	Control (Rate of patients, Median number of events)	ITT (Rate of patients, Median number of events)	Difference	PP (Rate of patients, Median number of events)	Difference
*Resources use*					
Hospital unplanned readmissions^a^	Rate = 35.5%, M = 2 £1,920.55	Rate = 12.9%, M = 1 £348.95	£1,571.60	Rate = 11.1%, M = 1 £300.26	£1,620.29
Emergency Department visit^b^	Rate = 54.8%, M = 2 £178.65	Rate = 54.8%, M = 1 £89.32	£89.33	Rate = 51.8%, M = 1 £84.43	£94.22
Unplanned GP consultation^c^	Rate = 48.4%, M = 1 £19.24	Rate = 25.8%, M = 1.5 £15.38	£3.86	Rate = 22.2%, M = 1 £8.82	£10.42
Total monetary	£2118.44	£453.65	£1,664.79	£393.51	£1,724.93
*MOC direct cost per patient*					
Cost of MOC^d^	0	£60.64^f^	(£60.64)	£59.25	(£59.25)
Cost of screening and recruiting^e^	0	£19.71	(£19.71)	£19.71	(£19.71)
*Summary Benefit–cost–analysis*					
Benefit–Cost Ratio with screening cost		20.72		21.85	

^a^average cost per night of hospital stay = £541, Median LOS for each readmission = 5 day, cost per readmission = £2,705 (541*5)

^b^average cost per visit = £163

^c^average cost per contact = £39.75

^d^Pharmacist time, hourly rate = £28.44 (senior pharmacist, band 8a). Estimated time for each patient; 1 h actual Clinic, 20 min pre-clinic preparation (0.33 h), post-clinic 15 min. Estimated time for the second intervention 30 min. Total time per patient = 125 min = 2.08 h (i.e. £59.25 per patient)

^e^Pharmacist time, hourly rate = £19.71 (newly qualified pharmacist, band 6). Estimated time to recruit one patient at least (1 h)

^f^Based on attendance disruption rate (4 patients were recruited but did not attend while 27 patients were recruited and attended) and the cost impact of such rate on the clinic will be as follows (4/27) *(0.33/2.08) = 2.35%; the ITT costs 2.35% more than PP

HRQOL (EQ-5D-3L)

At baseline in the management of usual activity and pain domains, seven (22.6%) and five patients (16.1%) respectively in the intervention group reported extreme problems, while in the control group the comparative data were three (9.7%) and four patients (12.9%) respectively. For the intervention group, improvement was noted at both the 30-day and 90-day assessment points for these parameters. Only one intervention patient reported extreme problems with usual activities and no patients reported extreme pain at the 90-day assessment.

In contrast, control patients exhibited a decline in their EQ-5D-3L scores, e.g. the number of patients who reported extreme problems was doubled at the 30-day assessment. Moreover, in the control arm four patients reported severe anxiety at the 30-day post-discharge assessment, while no intervention patients were in this category. Due to the small sample size, statistically significant differences between the intervention and control groups were seen only in the pain domain at the 30 day assessment (3.7% vs 27.6%; *P* = 0.026) and at the 90 day assessment (0% vs 23.1%; *P* = 0.01).

**Fig. 4:**
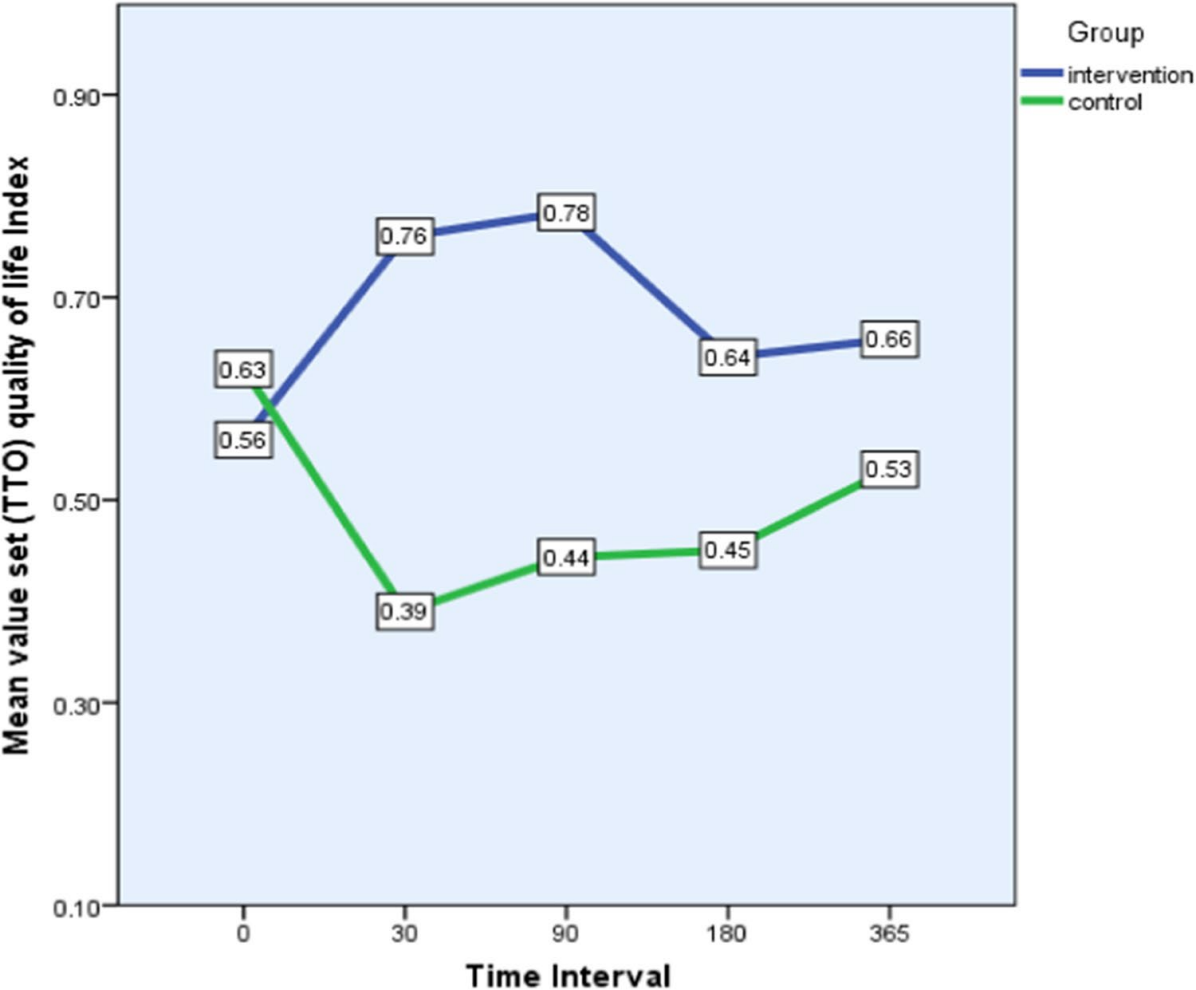
Within group and between group analysis of health-related quality of life index

Figure 4 shows the mean index (calculated using the time-trade-off technique) for the intervention and control groups at different time points. In general, the data indicate that the HRQOL index improved post intervention in the intervention group, but initially declined in the control group. There were no statistically significant differences at baseline (*P* = 0.16) and at 365 days (*P* = 0.15); differences were, however, statistically significant at the 30-day (*P* < 0.001), 90-day (*P* < 0.001) and 180-day (*P* = 0.036) time points.

### Patient beliefs about medicines questionnaire (BMQ)

Data collected using the beliefs about medicine questionnaire at baseline showed no statistically significant differences between groups. Intervention patients showed notable benefits in relation to their concerns about medicines at the 30-day assessment point, which was maintained throughout the study. The highest impact of the pharmacist intervention, on patient beliefs about medicines, was detected in two statements within the concern scale (I sometimes worry about the long-term effects of my medicines and my medicines are a mystery to me), with favourable mean differences for the intervention group, being 61.5% (*P* = 0.001) and 48.7% (*P* = 0.001) respectively.

**Fig. 5:**
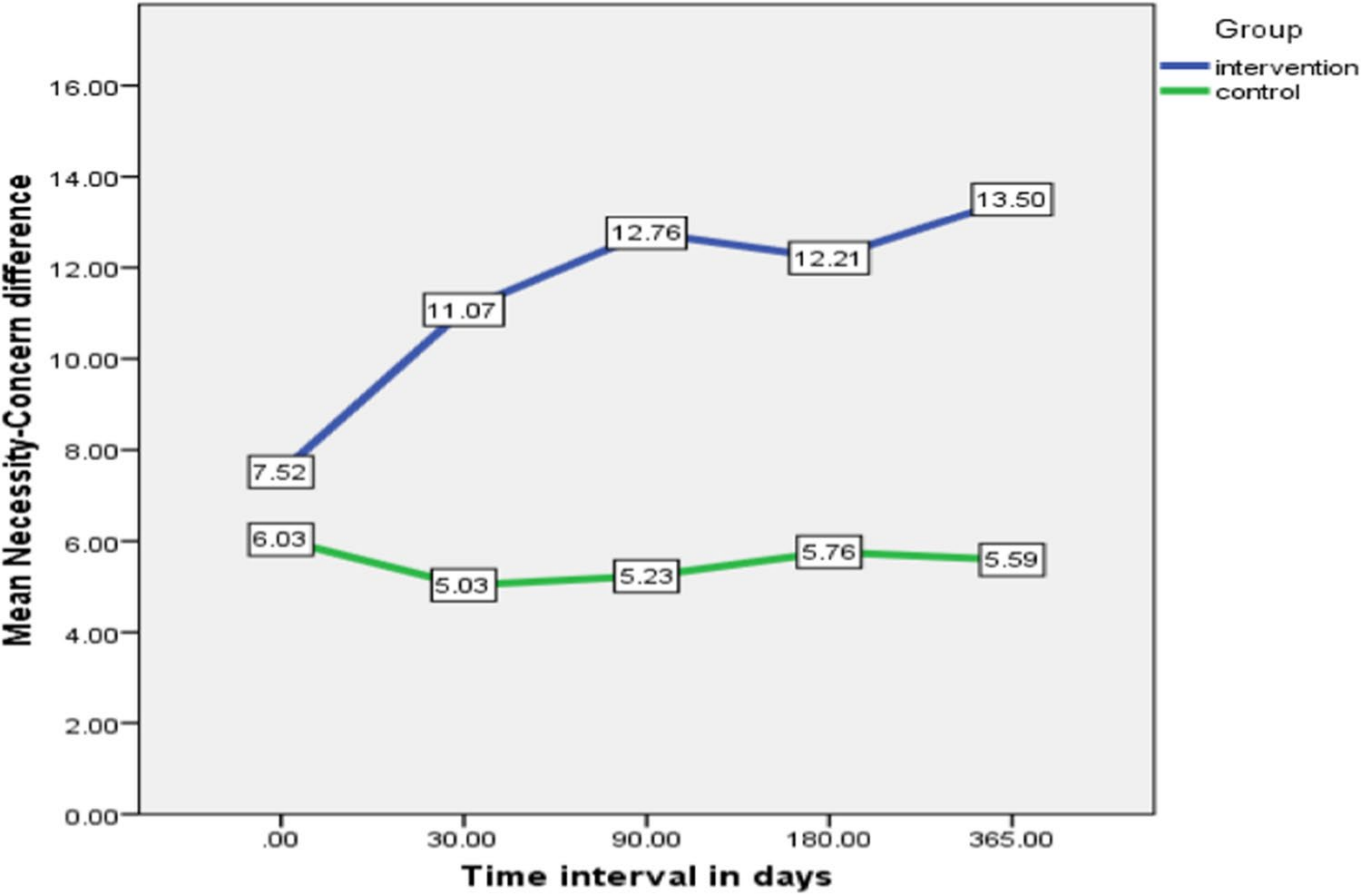
Within group and between group analysis of necessity-concern differential of BMQ

Figure 5 shows the calculated mean difference between the necessity and the concern scale for both intervention and control groups. After baseline, intervention patients showed significant improvement in their necessity-concern differential compared with the control group. Statistically significant differences were demonstrated at the 30-day (*P* < 0.001), 90-day (*P* < 0.001), 180-day (*P* < 0.001) and 365-day (*P* < 0.001) assessments.

Within group differences for the necessity-concern differential were significant for the intervention group (*P* = 0.018), but not for the control group (*P* = 0.23). Post hoc pairwise analysis within group indicated statistically significant differences between baseline and the following time points: 90-day (*P* = 0.024) and 365-day (*P* = 0.001), although there was a general trend of improvement across the complete follow-up period.

### Medication adherence report scale (MARS)

**Fig. 6:**
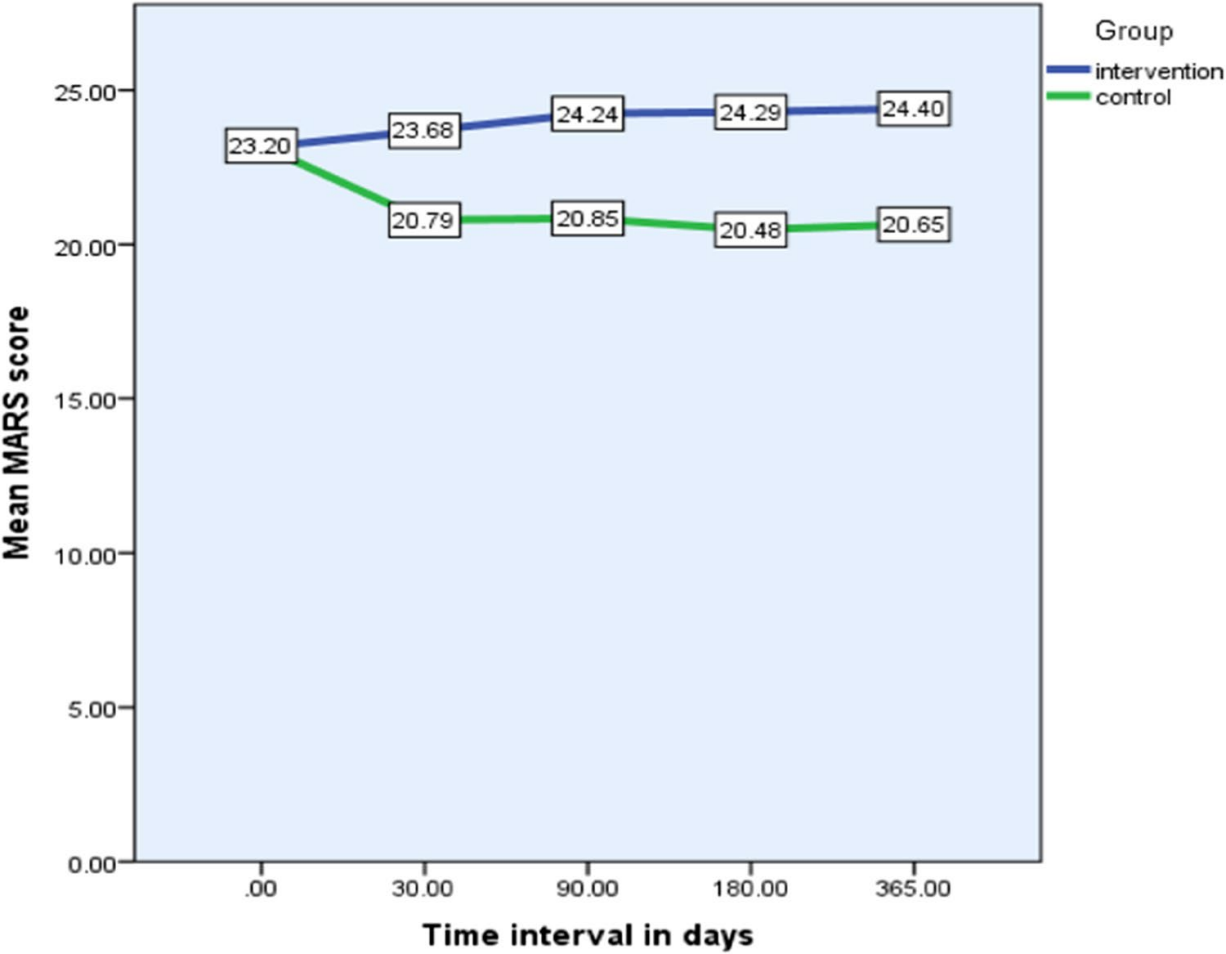
Within group and between group analysis of Medication Adherence Report Scale responses

Mean medicine adherence report scale scores exceeded 20 out of 25 throughout the study period in both control and intervention patients, which indicates acceptable adherence. There was a slight positive differential between and within groups (Fig. 6). Although some of the comparisons reached statistical significance, it is clear from the mean data that acceptable levels of adherence were evident throughout the study in both groups.

### Patient satisfaction

A bespoke patient satisfaction evaluation was posted to the PP patient group after receiving the MOC intervention. A total of 24 patients completed the questionnaire (response rate 88.9%). It was clear that the services were appreciated by patients, with only two patients being uncertain on one aspect, i.e. whether the number of clinic appointments was sufficient.

## Discussion

The recommended standard method for analysis of data collected in a RCT is the ITT approach as emphasised by the CONSORT guidelines [20, 21]. Nonetheless, this analysis has a number of drawbacks [22]. In the present study due to a fixed recruitment period, the work should be considered a pilot study rather than a definitive study. Since the intention was to determine the efficacy of the MOC intervention, PP analysis was carried out alongside an ITT analysis.

In the present study, a reduction in the 30-day readmission rate was noted (16.1% reduction in the PP assessment and a 9.6% reduction in the ITT assessment). Since this is the first evaluation of a new model of care delivery, there were no published data with which to compare the results. These data, however, compare favourably with a study in which pharmacist-led face-to-face discharge and telephone follow-up resulted in a 30-day readmission rate reduction of 14.2%; *P* = 0.01 [23]. In another study, pharmacist participation in follow-up appointments had a positive impact and led to a significant reduction (10.2%; *P* = 0.023) in 30-day readmission rates [24], where the control group involved matched patients from the previous year.

This study showed that clinic attendance decreased both ED visits and unplanned GP consultations. In a recent systematic review, which included 19 randomised controlled trials [25] it was estimated that in-hospital clinical pharmacist-led services (e.g. medicine review, medicine reconciliation, adherence assessment, therapeutic education, discharge counselling) resulted in a significant 30% reduction (risk ratio was 0.70, 95% CI 0.59–0.85, *P* = 0.001) in emergency department visits post-discharge. It should be noted, however, that many of these latter components are already provided in the Integrated Medicines Management (IMM) programme [7–9] that is delivered as routine practice to patients in the study site hospital by clinical pharmacy staff, i.e. the present results demonstrate additional authenticity to the IMM program. Variance in rates will exist due a greater throughput of older more comorbid patients who may not all have received the IMM service.

The positive impact of the provision of pharmacist-led interventions on HRQOL has been demonstrated by other researchers in a range of studies, utilising both generic and specific instruments [26–29]. A number of studies employed the EQ-5D tool. Some of the later studies confirmed the positive impact of pharmacist interventions [30, 31], while a number of studies reported no significant impact [32, 33]. In a recent systematic review [34] the authors concluded that pharmacist care can significantly improve at least one dimension of HRQOL, usually the pain related dimension, i.e. similar to the present findings.

Improvements in beliefs about medicines as a result of pharmacist interventions are consistent with previously published studies [35, 36]. However, the present research showed that the core improvement was realised via the concern scale rather than the necessity scale. The maximum impact was seen within 90 days but continued impact was observed at 12-months post-discharge. The results for patient scores in the MARS indicated that self-reported adherence levels were high across both groups over the study period. The level of patient satisfaction with the MOC was generally high indicating the high acceptability by patients of extended post-discharge services noted by others [37–39].

This is the second proposed model of post-discharge care put forward by this research team; the other being a post-discharge telephone follow-up [40]. The need for both services exist because some patient/problems need a face-to-face interaction in order that an appropriate solution can be achieved. More work is required in this area to determine the criteria that would direct patients to the most appropriate service. Further work could also incorporate a comparison of the hospital-based model versus the community pharmacy based Ensing model to determine which would be more cost effective [41–45].

### Limitations

The sample size was much smaller than originally planned, thus reducing the statistical power of the study. Nonetheless, the study provided valuable proof of concept pilot data, which can be built upon in future work. Secondly, the study was a single centre trial so results may not be generalisable. The design of the study restricted recruitment to patients who managed their own medicines.

## Conclusion

The implementation of a pharmacist-led MOC post-discharge had a positive impact on unplanned readmissions, multiple readmissions, utilisation of ED visits and GP consultations. Positive cost–benefit results and patient centered humanistic outcomes including beliefs about medication, HRQOL and satisfaction resulted from the intervention. This novel approach to post-discharge care should be examined in a large, multi-centre trial to evaluate its impact on patient care.
